# Light-at-night, cancer and aging

**DOI:** 10.18632/aging.100126

**Published:** 2010-03-04

**Authors:** Christian Bartsch

**Affiliations:** Center for Research in Medical and Natural Sciences, University of Tübingen, D-72074 Tübingen,Germany

**Keywords:** LAN, cancer, aging, circadian clock, melatonin

Recently, the International Agency for Research on Cancer (IARC) concluded that  "shift-work that involves circadian disruption is probably carcinogenic to humans" [1]. This conclusion was based upon limited evidence in humans [2,3] and sufficient evidence in experimental animals [4,5]. The current experimental results of Vinogradova and colleagues from Petrozavodsk and St. Petersburg render further support to this important issue and indicate a number of highly relevant points that will have to be taken into consideration for a better understanding of the mechanisms involved and possible extrapolations to the situation in humans. Their aim was see in which way constant bright light of 750 Lux (LL) may affect survival and tumor development of normal outbred male and female rats of the LIO-strain in comparison to animals kept under a standard photoperiod of LD=12h:12h. LL was applied life-long starting from either 1 or 14 months of age. At one month of age animals are becoming sexually mature whereas at 14 months sexual competence declines. The authors found that dramatic life-shortening effects are observed among female rats, particularly in those where LL-treatment was started at one month of age. They interpret these findings to indicate that constant light probably exerts its detrimental effects on health, tumorigenesis as well as survival via disturbances of the female reproductive cycle [6]. The mechanisms involved can be assumed to include the pineal hormone melatonin as well which as chemical signal of darkness and controlled by the central circadian clock in the N. suprachiasmatici [7] may play a very central part since it is suppressed by LL and participates in the neuroendocrine control of the female reproductive system. In addition, the authors present interesting data that disturbances in the regulation of the anti-oxidative enzymes superoxide-dismutase and catalase due to LL are also involved. These enzymes are known to be controlled by melatonin due to its anti-oxidative action [8]. These fascinating results of the team led by V.N. Anisimov clearly show that for a better understanding of the highly relevant issue of shift-work but also of jet-lag and space-flight and other life-styles connected with circadian disruption [9-12] it will be important to specifically consider developmental aspects. It appears to be very clear that photoperiodic experiences early in life are essential determinants for carcinogenic processes to develop at higher age. An important question to be addressed by future experiments is whether there might be a critical period towards the negative effects of LL in and around early adulthood or whether circadian disturbances have to persist over a longer period of time throughout life to promote cancer. We have to be thankful to the authors that they have started to open our eyes to consider such basic and complex issues and it will have to be seen in which way these findings will be applicable to women who are increasingly afflicted by breast cancer.
